# Older Adult User Feedback on Design and Functionality of EngAGE, a Voice-Activated Exercise Program

**DOI:** 10.1093/geroni/igab046.1024

**Published:** 2021-12-17

**Authors:** Roscoe Nicholson, Yadira Montoya, Saira Shervani, Chelsea Smith, Louise Hawkley, Megan Huisingh-Scheetz

**Affiliations:** 1 University of Chicago, Evanston, Illinois, United States; 2 NORC at the University of Chicago, Chicago, Illinois, United States; 3 University of Chicago, Chicago, Illinois, United States

## Abstract

The EngAGE Alexa app is a socially motivated exercise program targeting older adult-caregiver dyads to promote mobility independence. EngAGE provides exercise routines that older adults can perform in the home in conjunction with a messaging component to facilitate motivation from caregivers and a tracking component to monitor progress. This presentation will describe the qualitative results that have informed the app’s design and evaluation of its feasibility and functionality following a 14-week feasibility study in 10 dyads of older adult exercisers and their caregivers. The presentation will cover the perceived benefits of EngAGE’s older adult users (including “real world” clinically relevant improvements, the comprehensiveness of the exercises, and exercise knowledge gained), as well as likes and dislikes that contributed to our assessment of the app’s functionality. Finally, we will discuss how the feedback contributes to future directions in the development of the app’s features, supporting materials, design and content.

